# Role of Mass Transport in Electrochemical CO_2_ Reduction to Methanol Using Immobilized Cobalt Phthalocyanine

**DOI:** 10.1021/acsaem.3c02979

**Published:** 2024-04-04

**Authors:** Thomas Chan, Calton J. Kong, Alex J. King, Finn Babbe, Rajiv Ramanujam Prabhakar, Clifford P. Kubiak, Joel W. Ager

**Affiliations:** † Liquid Sunlight Alliance, Lawrence Berkeley National Laboratory, Berkeley, California 94720, United States; ‡ Chemical Sciences Division, Lawrence Berkeley National Laboratory, Berkeley, California 94720, United States; § Materials Sciences Division, Lawrence Berkeley National Laboratory, Berkeley, California 94720, United States; ∥ Department of Materials Science and Engineering, University of California, Berkeley, Berkeley, California 94720, United States; ⊥ Department of Chemical and Biomolecular Engineering, University of California, Berkeley, Berkeley, California 94720, United States; # Liquid Sunlight Alliance, University of California, San Diego, La Jolla, California 92093, United States; ¶ Department of Chemistry & Biochemistry, University of California, San Diego, La Jolla, California 92093, United States; ∇ Department of Nanoengineering, University of California, San Diego, La Jolla, California 92093, United States

**Keywords:** CO_2_ reduction, multiwalled carbon nanotubes, catalysis, methanol selectivity, mass-transport

## Abstract

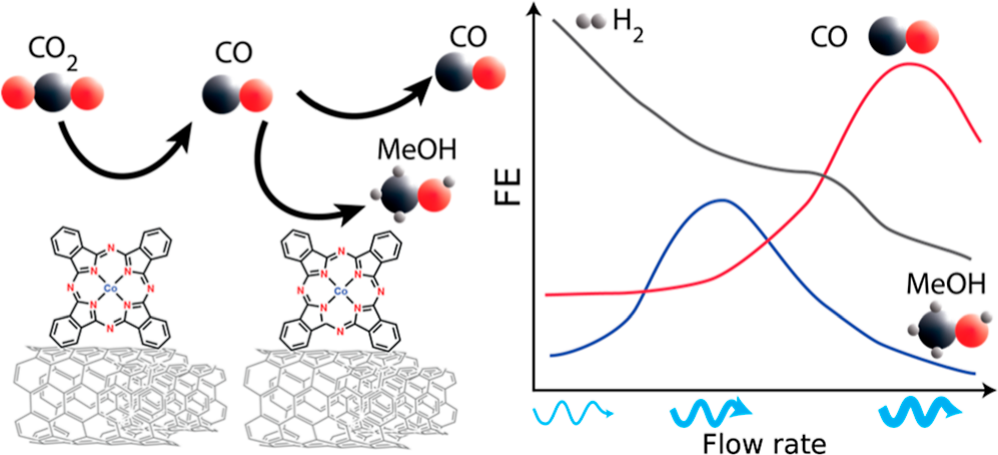

Electrochemical CO_2_ reduction (CO_2_R) using heterogenized molecular catalysts usually yields 2-electron reduction products (CO, formate). Recently, it has been reported that certain preparations of immobilized cobalt phthalocyanine (CoPc) produce methanol (MeOH), a 6-electron reduction product. Here, we demonstrate the significant role of intermediate mass transport in CoPc selectivity to methanol. We first developed a simple, physically mixed, polymer (and polyfluoroalkyl, PFAS)-free preparation of CoPc on multiwalled carbon nanotubes (MWCNTs) which can be integrated onto Au electrodes using a poly(3,4-ethylenedioxythiophene) polystyrenesulfonate (PEDOT:PSS) adhesion layer. After optimization of catalyst preparation and loading, methanol Faradaic efficiencies and partial current densities of 36% (±3%) and 3.8 (±0.5) mA cm^–2^, respectively, are achieved in the CO_2_-saturated aqueous electrolyte. The electrolyte flow rate has a large effect. A linear flow velocity of 8.5 cm/min produces the highest MeOH selectivity, with higher flow rates increasing CO selectivity and lower flow rates increasing the hydrogen evolution reaction, suggesting that CO is an unbound intermediate. Using a continuum multiphysics model assuming CO is the intermediate, we show qualitative agreement with the optimal inlet flow rate. Polymer binders were not required to achieve a high Faradaic efficiency for methanol using CoPc and MWCNTs. We also investigated the role of formaldehyde as an intermediate and the role of strain, but definitive conclusions could not be established.

## Introduction

Electrochemical CO_2_ reduction (CO_2_R) is of interest for sustainable chemical and fuel production.^1,2^ Molecular catalysts, such as cobalt phthalocyanine (CoPc) and fac-Re(2,2′-bipyridine)(CO)_3_Cl, whether in homogeneous form or immobilized onto a surface, have shown high CO_2_R activity and selectivity toward 2e^–^ reduction products, CO and formate.^3−8^ The effects of molecular catalyst immobilization/heterogenization onto conductive supports such as carbon nanotubes (CNTs) or polyaniline has been extensively investigated.^4,5,9−21^ In the case of CoPc, some immobilization strategies have been very effective, enabling integration gas diffusion electrodes which can be operated at >100 mA cm^–2^ with almost 100% Faradaic efficiency (FE) to CO.^22,23^

Recently, there have been reports that immobilized CoPc can directly reduce CO_2_ to methanol, a 6e^–^ product, in aqueous electrolytes.^17,24−27^ Boutin et al., using mixed suspensions of CoPc and CNTs, reported relatively low MeOH FEs (<2%), with higher values being achieved when CO was used as the feedstock.^25^ The study of Wu et al. shows that the catalyst dispersion method is important; by dissolving CoPc in DMF (as opposed to forming a suspension), FEs to MeOH of 44% were achieved.^24^ Su et al. further demonstrated that biaxial strain of the planar CoPc molecule over different sized nanotubes greatly affects the MeOH selectivity, with highly strained CoPc on small CNTs leading to MeOH FEs greater than 50%.^17^ Although not mentioned in Wu’s study, we posit that the CoPc could also have been significantly strained as MWCNTs with diameters ranging from 10 to 15 nm were used. We further note that these studies used Nafion as a polymer binder, which is not only potentially environmentally problematic but also introduces other factors, such as additional electrical resistance, which are convoluted with the CoPc activity.

While there is general agreement that CO* is an important intermediate, the mechanism for CO_2_R to MeOH on CoPc/CNTs is not fully elucidated. Moreover, CO desorption from CoPc is known to be energetically favorable, so the notion that it is an intermediate to MeOH is somewhat counterintuitive.^17,28−30^ However, it has been suggested that at very negative potentials, formation of the formyl (OCH*) intermediate becomes favorable compared to CO desorption.^30^ The MeOH selectivity with increased CoPc tensile strain and better dispersion are similarly rationalized in terms of decreased favorability of CO desorption; for strain, lowering the formyl formation barrier is identified as an additional factor.^17^ Still, there has been disagreement as to whether the formyl intermediate can lead to formaldehyde.^28,31^ For example, two studies by Boutin and co-workers provided evidence that the formyl adduct is reduced to MeOH but can also desorb as formaldehyde, some fraction of which could be further reduced to MeOH.^25,31^

The central aim of this study is to critically examine the role of the CO intermediate in CO_2_R to MeOH on CoPc/MWCNT catalysts. We hypothesized that in a liquid flow cell environment, if CO is a mobile intermediate, the flow rate of CO_2_-saturated aqueous electrolyte should affect the relative selectivities for CO and MeOH, noting that a similar methodology has been used to investigate the role of CO in CO_2_R on Cu.^32^ We were further motivated to eliminate PFAS-containing materials from the preparation of CoPc/MWCNT catalysts and describe here a simple, physically mixed, polymer-free preparation of immobilized CoPc on MWCNTs adhered to a Au substrate using a PEDOT:PSS layer. By removing all polymer binders from the catalyst ink, we isolate just effects due to interactions of the CoPc with the MWCNTs. Furthermore, this layered structure of CoPc/MWCNT on PEDOT:PSS on Au could enable future integration onto solar cells.^33^ Since the catalyst preparation is new, we first optimized the loading, a parameter that has been explored extensively for CO_2_R to CO but not MeOH, and investigated the effect of applied reductive potential. Next, we demonstrate that changing the electrolyte flow rate (and thus transport properties) changes the selectivity toward MeOH, CO, and H_2_, with an intermediate flow rate showing optimal MeOH selectivity. Multiphysics simulations are in good agreement with the experimental data and emphasize the finding that balancing the rates of CO consumption and transport is important for MeOH selectivity. Interestingly, we observed formation of aggregates, showing that uniform dispersion is not necessary for selective CO_2_R to MeOH. Finally, we detected formaldehyde in some experiments, hinting that it might play a role in the mechanism, but a definitive picture could not be obtained. Similarly, because the electrochemically active fraction of CoPc is relatively small in our optimal preparation, we could not definitively evaluate the role of strain.

## Experimental Section

### Materials

Except for multiwalled carbon nanotubes (MWCNTs), all materials were used as purchased without further purification. Purchased catalyst materials include 12 nm × 10 μm MWCNTs (98% Sigma-Aldrich) and cobalt(II) phthalocyanine (ThermoFisher). Dispersions were made in isopropyl alcohol (IPA) (Sigma-Aldrich ≥99.5%). 3–4% (wt, in H_2_O) high conductivity PEDOT:PSS (>200 S/cm, Sigma-Aldrich) was used as an adhesion layer. MWCNTs were purified in HPLC grade HCl (Sigma-Aldrich 99.999% purity) to avoid introducing metal impurities. Formaldehyde (37 wt % in H_2_O) and its derivatizing agent pentafluorobenzylhydroxylamine (PFBHA) were purchased from Sigma-Aldrich. Carbon fiber paper Toray 120 (Fuel Cell Store) was used as the anode and a SELEMION membrane (AGC Engineering) was used to separate the anode and cathode chambers of the electrochemical cell. Potassium carbonate (99% purity, Sigma-Aldrich) and potassium bicarbonate (99.7% purity, Sigma-Aldrich) were used in electrolyte preparation.

### Catalyst Ink Preparation

MWCNTs were purified by sonication in 6 M HCl for 1 h in a sonication bath. The suspension was then stirred with a Teflon stir bar on a hot plate for 23 h followed by filtration through a fritted funnel. The MWCNTs were rinsed with MiliQ pure deionized water until the effluent water reached a neutral pH. The MWCNTs were then dried on a high vacuum Schlenk line overnight. Inks with differing ratios of MWCNTs to CoPc were made and used immediately. Most of the experiments were performed with a 1:0.6 ratio, which was made by weighing 20 mg of MWCNTs and 12 mg of CoPc in a centrifuge tube, followed by the addition of 32 mL of IPA. The resultant suspension was probe sonicated (Cole-Parmer 500 W probe sonicator) at 37% amplitude for 1 h which imparts approximately 89 kJ into the ink. An ice bath was used to keep the ink cool and to prevent the solvent from evaporating. Different ratios of catalyst to MWCNTs were used in other loadings, but the procedure remained the same: a ratio of 1 mg of material to 1 mL of IPA was maintained throughout. Smaller batches used 30 min of sonication instead of 1 h.

### Electrode Preparation

#### Glass Slide Preparation

2.5 cm × 2.5 cm slides were cut from larger glass slides using a diamond tipped scribe. They were then cleaned by bath sonication in the following solvents (in order for 10 min each): acetone, soap water, DI water, and IPA (12–20 at a time using a custom-made wafer holder).

#### Sputtering of Metals

An adhesion layer 10 nm of Ti was sputtered (AJA International Magnetron Sputtering) onto the glass slides at 150 W power and 3 mTorr Ar (deposition rate: 1.0 Å/s). 150 nm of Au was then sputtered at 150 W and 3 mTorr Ar (deposition rate: 2.94 Å/s). The targets were located off center with respect to the sample stage, which was rotated at 100 rpm. A quartz crystal monitor was used to measure the deposition rates.

#### PEDOT:PSS Layer

2.4 mL of 3.0–4.0% high conductivity PEDOT:PSS was diluted in approximately 10 mL of H_2_O. The ink was then sprayed onto 24 glass/Ti/Au slides using an ultrasonic spray coater (SonoTek ExactaCoat) at a flow rate of 0.1 mL/min and run power of 2 W, with the stage being held at 95 °C. The areal spray efficiency (sample area/spray area) is 80%, leading to each sample having ∼80 μL of the 3–4% PEDOT:PSS. This process can be easily scaled down for smaller batches.

#### Catalyst Layer

The catalyst ink was loaded into a hand-held Master G76 air brush and then sprayed onto the samples while they were heated on a hot plate set to 85 °C. The samples were weighed multiple times before and after spray steps to calculate the mass loadings of MWCNTs and CoPc.

### Electrochemical Testing

Electrochemical tests were performed with a custom flow cell, with a peristaltic pump (Cole-Parmer) used to control the electrolyte flow rate. CO_2_ was constantly sparged into the catholyte (0.1 M KHCO_3_) reservoir at 5 sccm during electrolysis. A leakless Ag/AgCl reference was used, and the cell was controlled with a potentiostat (BioLogic SP-300). IR-corrected chronoamperometry (CA) was performed for 80 min for each test. The gas stream was connected to a gas chromatograph (SRI Multi Gas 3) for gas quantification, and the liquid products were quantified by nuclear magnetic resonance. Formaldehyde was quantified by derivatization using PFBHA followed by GC–MS analysis (Agilent 7890A). Cell schematics and more experimental details in the Supporting Information.

### X-ray Photoelectron Spectroscopy

X-ray photoelectron spectroscopy (XPS) measurements were done on Kratos Axis Ultra DLD system using a monochromatized Al X-ray source. Samples were measured before and after electrochemical testing, with the probe area being the portion of the surface exposed to the electrolyte. High-resolution spectra were recorded in the spectral regions corresponding to the C 1s, N 1s, and Co 2p peaks. Spectral fitting was conducted using CasaXPS analysis software.

### Scanning Electron Microscopy

Scanning electron microscopy (SEM) imaging was performed on a FEI Quanta 250 SEM. The beam was set to 10 keV during the measurement.

## Results and Discussion

### Immobilization of CoPc on MWCNTs and Optimization of Loading Conditions

Integration of CO_2_R catalyst inks to metal substrates will enable integration with solar cells for photoelectrochemical applications. Au is a common solar cell back contact, motivating us to use it in this study. CNTs are known to have poor adhesion to Au and other materials without special preparation.^34^ Indeed, we found that electrodes made by simply spraying CNT-catalyst inks onto Au-coated substrates were not stable, with delamination occurring within 30 min of operation (gas evolution exacerbated the issue).

Molecular attachment methods using thiol anchors to Au have been employed for other types of electrocatalysis and for spectroscopy, but we did not expect them to be stable under the reducing surface conditions of CO_2_R.^19−21,35,36^ Instead, we modified the Au substrate by spray coating a thin layer of high conductivity poly(3,4-ethylenedioxythiophene) polystyrenesulfonate (PEDOT:PSS) atop the Au. We found this significantly improved adhesion and enables the CoPc/MWCNTs to form a uniform film atop the Au-coated substates.

We sought to find the catalyst loading, catalyst-to-support ratio, and potential window which could be used throughout the study, noting that prior reports have found that immobilized CoPc will form methanol in only a relatively narrow range of conditions. Boutin et al., using a similarly physically mixed CoPc/MWCNT catalyst, only reported trace amounts of MeOH at −0.88 V versus RHE, but did not explore more negative potentials or higher loadings.^25^ We hypothesized that higher catalyst loading and more negative potentials would increase the methanol selectivity. In tests performed at −1.2 V versus RHE, we found that, in general, catalysts with low CoPc:MWCNT ratios had low FEs for methanol (with significant variance) while larger ratios (0.6:1 and higher) led to better selectivity (>30% FE for methanol, Figure S3). A ratio of 0.6:1 CoPc:MWCNT, corresponding to a loading of 0.15 mg cm^–2^ CoPc had both good methanol selectivity and operational stability and was thus selected as the base case for this study.

To confirm that CoPc is responsible for the methanol production, we performed control experiments with Au, PEDOT:PSS/Au, and MWCNT/PEDOT:PSS/Au electrodes: none produced methanol. Also, there was little selectivity to CO (<20% FE, Figure S4), even at the very negative potential employed (−1.2 V vs RHE). Experiments performed with ^13^C labeling showed that the carbon source for the methanol was the supplied CO_2_ (Figures S6–S9). We also performed an experiment using CO as the gas feed which produced methanol (Figures S10 and S11).

### Optimal Potential for Methanol Formation

**Figure 1:**
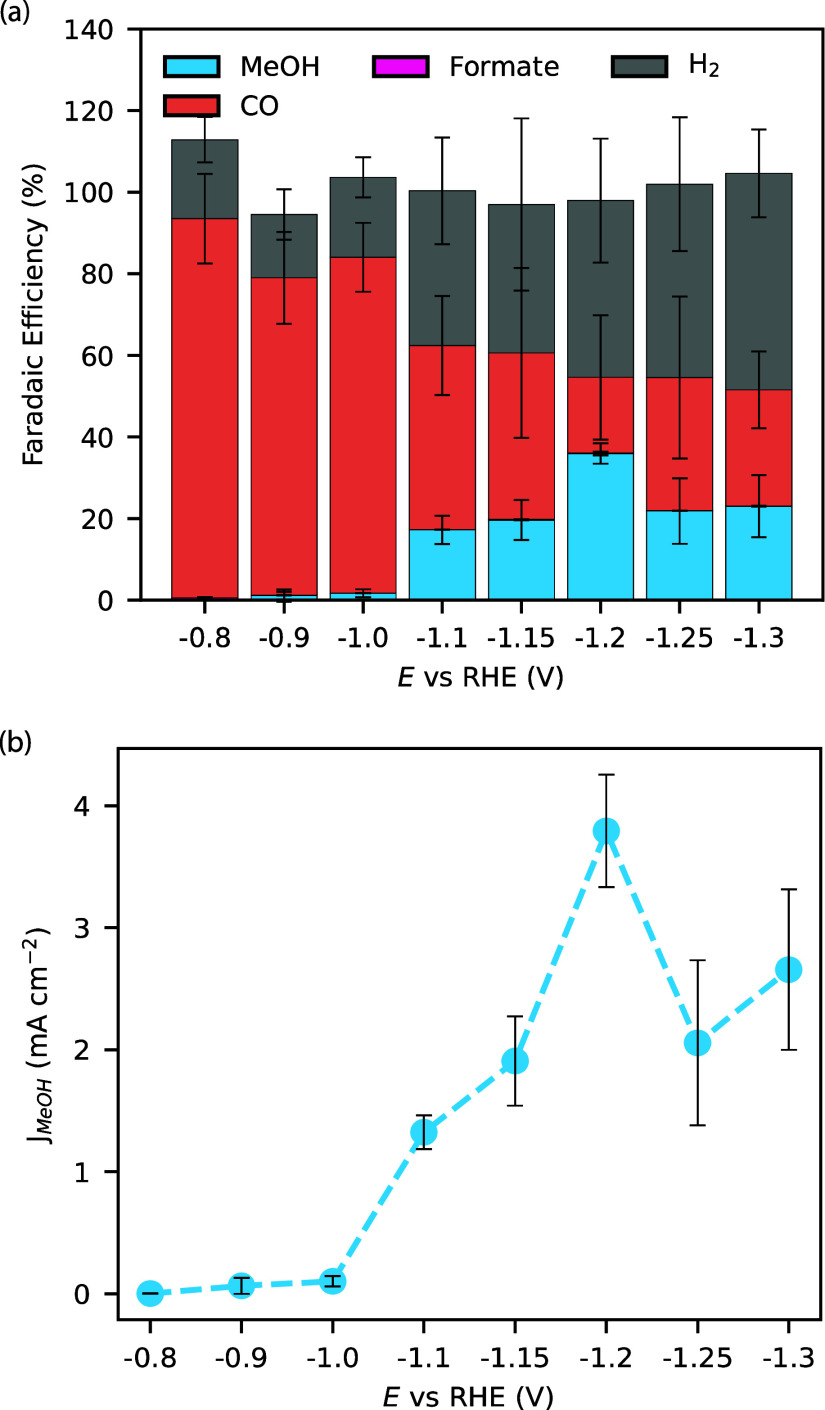
(a) FE and (b) partial (MeOH) current density as a function of potential. Flow rate, loading, and CoPc:MWCNT ratio, were 8.5 cm/min, 0.15 mg cm^–2^, and 0.6:1 respectively. Conditions were CO_2_-saturated 0.1 M KHCO_3_ electrolyte. Error bars are from 3 or more replicates.

Consistent with prior reports, we find that the methanol selectivity for immobilized CoPC is highly sensitive to the potential, Figure 1. Very small amounts of methanol are observed at −0.8 V versus RHE (<1% FE). Methanol selectivity increases at more negative potentials, reaching a maximum FE of 36% (±3%) at −1.2 V versus RHE (3.8 ± 0.5 mA cm^–2^ partial current density, see Figure S11 for total current densities). Both FE and methanol partial current density decline at still more negative potentials. We suspect that methanol formation is still possible at even more negative potentials (i.e., less than −1.3 V vs RHE), but we were not able to evaluate in this region due to catalyst film delamination caused by the high rate of gas bubble formation.

### Role of Mass Transport

**Figure 2:**
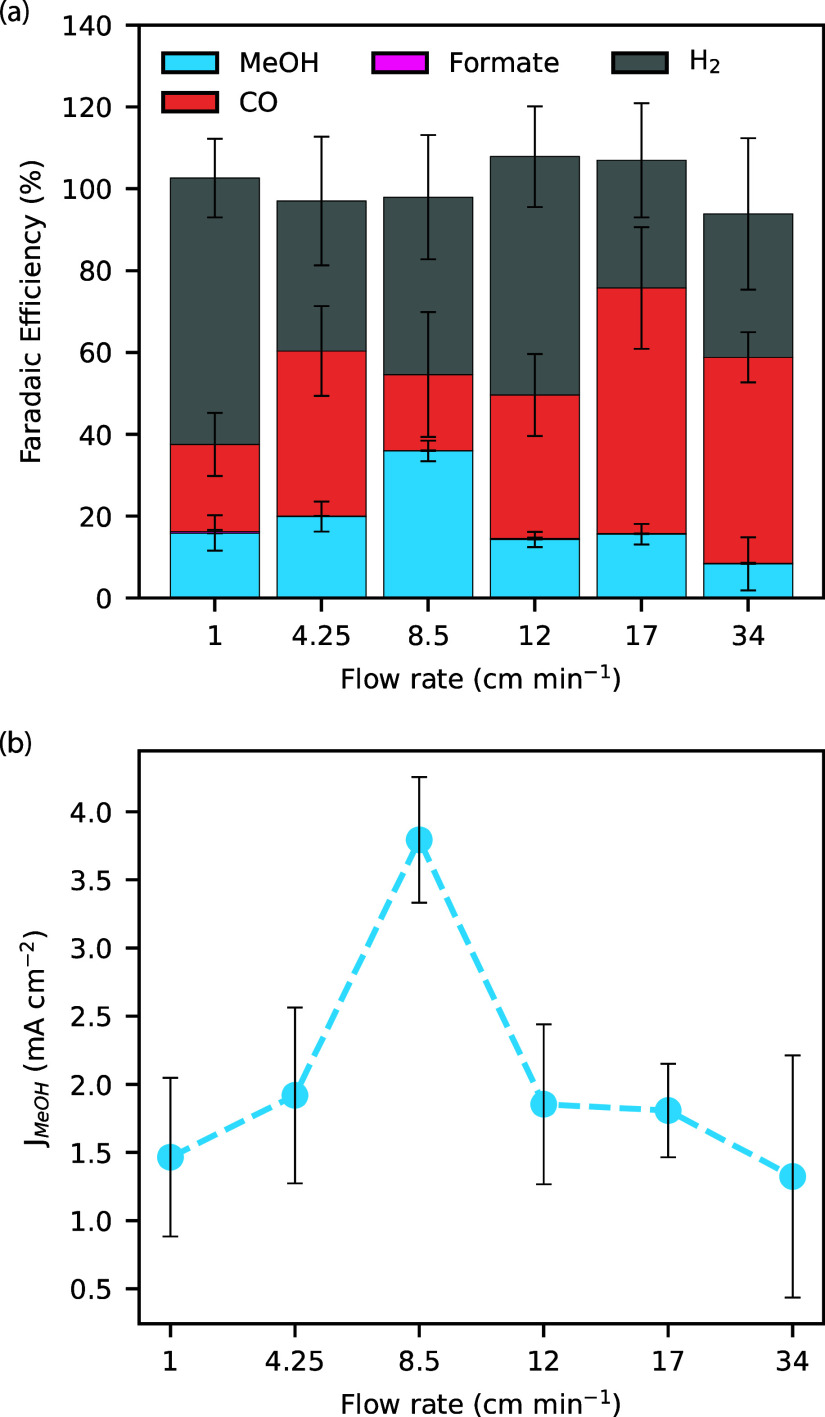
(a) FE for methanol as a function of flow rate (b). Partial current density of methanol as a function of electrolyte flow rate showing an optimal flow rate around 8.5 cm/min. Conditions are CO_2_-saturated 0.1 M KHCO_3_ electrolyte at −1.2 V vs RHE. Error bars are from three or more replicates.

The electrolyte flow rate strongly affects the selectivity of CoPc/MWCNT electrocatalysts, as shown in Figure 2. For example, both the methanol FE (Figure 2a) and partial current density (Figure 2b) peak at a flow rate of 8.5 cm/min, with significantly smaller values being observed at smaller and larger flow rates. There is also a clear trend in the H_2_ and CO selectivities with the former being favored at low flow rates, while the latter is favored at higher flow rates. CoPc/MWCNT material has been shown to preferentially reduce CO_2_ over hydrogen evolution reaction (HER), due to a high Co–H hydride formation energy.^37,38^ CO_2_ concentration profiles calculated from Multiphysics simulations (Figure S15) suggest that the surface is more depleted of CO_2_ at low flow rates, allowing HER to favorably compete.

The observation of increasing CO selectivity with increasing electrolyte flow rate suggests that CO can desorb from, and readsorb on, the CoPC/MWCNTs, with desorption being competitive with conversion into methanol. That is, at high electrolyte flow rates, the CO intermediate is swept away before it can be converted. Therefore, we hypothesize that for selective methanol generation, there needs to be a balance between mass transport of the intermediates and local consumption and concentration of these intermediates.

**Figure 3:**
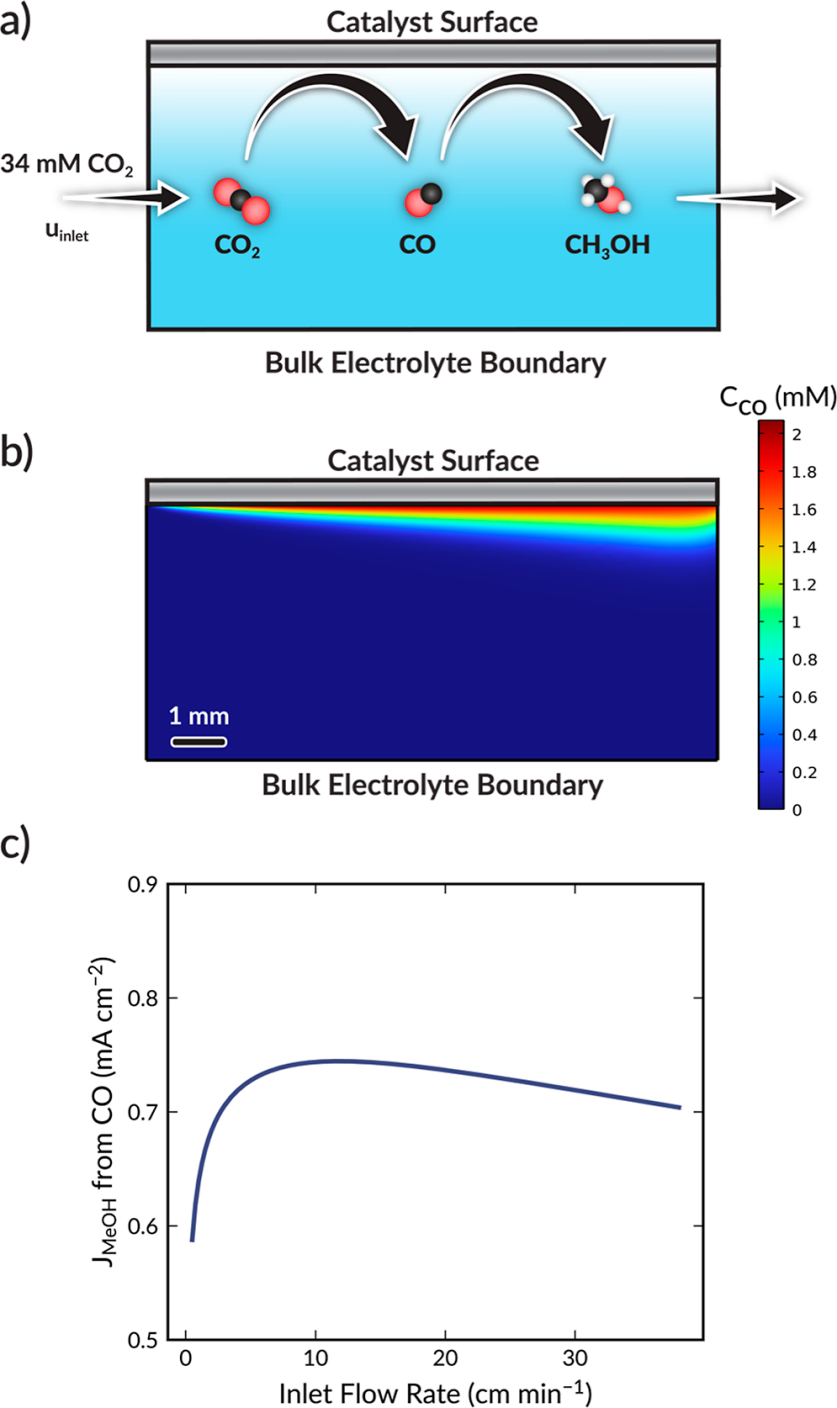
(a) Schematic illustration of 2D continuum model and cascade chemistry. (b) Surface plot of CO concentration throughout the model domain at an inlet flow rate of 8.5 cm min^–1^. (c) Simulated methanol current density (*j*_MeOH_) from CO reduction as a function of inlet flow rate.

To explore this rationale, a 2D continuum model of the electrolyte boundary layer adjacent to the cathode surface was developed, which is illustrated in Figure 3a. The model simulates the transport of CO_2_ and CO via convection and diffusion and the simultaneous generation and consumption of CO across the cathode; further modeling details are provided in the Supporting Information.

The predicted CO concentration profile at an inlet flow rate of 8.5 cm min^–1^ is presented in Figure 3b. At ≳8.5 cm min^–1^, convection is the dominant mode of transport and helps to keep CO localized to the cathode surface. However, beyond ∼10 cm min^–1^ the rapid rate of convection sweeps much of the CO out of the system before it can be reduced (see Figure S14). Below 8.5 cm min^–1^, CO diffuses away from the cathode into the bulk of the electrolyte, as seen in Figure S14a, rather than converting into methanol. The resulting impact of inlet flow rate on CO reduction to methanol is seen in Figure 3c, which shows a peak in the predicted methanol current density at ∼10 cm min^–1^. Thus, the model supports the hypothesis that inlet flow rate changes CO_2_R product selectivity by modulating the transport of unbound intermediates. We note that the methanol current density predicted by the model is lower than that measured experimentally because the model neglects the heterogeneity of the catalyst (i.e., it assumes a planar, flat catalytic surface, when experimentally the catalyst is a porous layer). Nonetheless, the simulation still predicts an optimal inlet flow rate for producing methanol, which matches the experimental findings.

### Role of Strain and Aggregates

Properties such as strain and aggregates are known to affect CoPc behavior on carbon nanotubes, with dissolution of CoPc facilitating CoPc conformation over small diameter MWCNTs or SWCNTs leading to biaxial strain that improves MeOH selectivity.^17,24,39,40^ Aggregates can also form because π–π stacking interactions between CoPc molecules are stronger than those between CoPc and the CNT. Previous experimental reports on strain and aggregates have suggested that both strain and absence of aggregates are necessary for selective CO_2_R to MeOH.^17,24^ One theory study suggested that CO desorption is generally more favored in CoPc dimers (the simplest “aggregate”) compared to a monomeric dispersion.^30^ It also suggests that formation of a formyl intermediate in aggregates might compete with desorption at more significant reducing potentials, but at which HER dominates.

**Figure 4:**
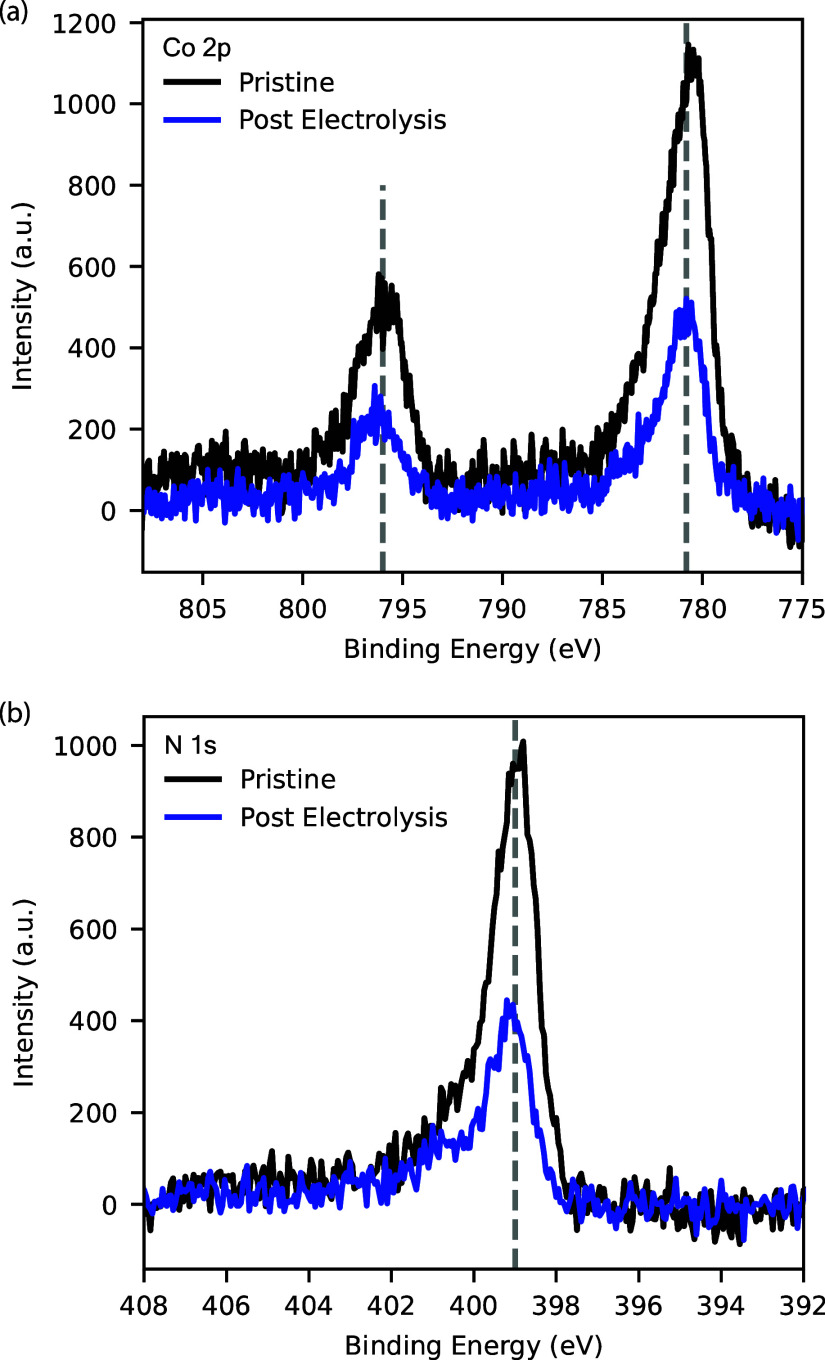
XPS core level spectra for (a) Co 2p and (b) N 1s for a CoPc/MWCNT electrode surface before and after CA @ −1.2 V vs RHE. The dashed lines indicate that the N 1s and Co 2p energies do not change after electrolysis.

**Figure 5:**
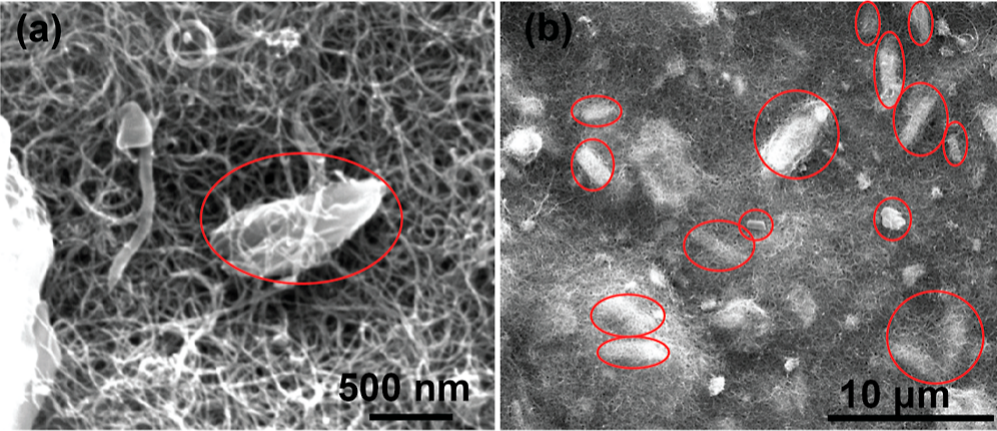
SEM image of the pristine CoPc/MWCNT electrode surface (loading 0.15 mg cm^–2^) showing high levels of CoPc crystallite aggregates (a) at 50 kX and (b) at 5 kX magnification. Red circles show locations of CoPc aggregation.

To determine if our catalysts were strained, we performed XPS measurements on the CoPc (Figure 4). XPS spectra for N 1s of strained CoPc appears as two overlapping peaks, with increasing strain increasing the energy difference as was previously reported.^17^Figure S16 shows an XPS spectrum of strained CoPc prepared on small single-walled CNTs. For our CoPc/MWCNTs, we observe a single N 1s peak, which could indicate that our catalyst is unstrained. The Co 2p energies are also consistent with an unstrained catalyst. However, SEM images in Figure 5 clearly shows CoPc aggregate formation prior to operation and the CV scan of Co(II)/Co(I) redox feature indicates an electroactive fraction of <10% (Figure S17). For these reasons, direct interpretation of the XPS spectrum is difficult, as the inactive (and presumably unstrained) aggregates could be dominating the signal, and we cannot make definitive statements regarding the role of strain. In contrast, because the CoPc/MWCNTs in this study clearly contain aggregates but still have comparable MeOH selectivity to prior reports, we find that dispersion (via CoPc dissolution), on its own, is not a main determinant of MeOH selectivity on CNTs as was suggested in a previous report.^24^ On the other hand, other factors such as loading, potential, and CO transport greatly influence selectivity.

### Limitations of the Study and Future Scope

The role of formaldehyde (four electron reduction products) in CO_2_R on CoPc is also still unresolved. In general, formaldehyde is under/unreported product in the CO_2_R literature, partly because its analytical chemistry is more challenging compared to other liquid products. There are competing thoughts as to whether formaldehyde is an intermediate, with one claiming and measuring formaldehyde’s existence and another claiming that formaldehyde desorption is too energetically unfavorable.^28,31^ This led us to originally hypothesize that if formaldehyde were indeed an unbound intermediate, we should observe an increase of formaldehyde FE with flow rate similar to what was shown for CO in Figure 2a. Our experimental results were insufficiently consistent to support this hypothesis. Some runs led to formaldehyde concentrations in the electrolyte of up to 0.1 mM, corresponding to about 0.5% FE. Figure S18 shows an example chromatogram and mass spectrum of a sample containing formaldehyde. However, some runs did not produce detectable amounts of formaldehyde. We speculate that formaldehyde is either converted very quickly and/or has a different transport path compared to CO, but further study is clearly needed. Quantitative measurement of formaldehyde might also be a challenge due to outgassing; use of temperature-controlled cells may be required.

Our finding that CO is an important, but unbound intermediate hints at competition between CO_2_ and CO for binding sites. We hypothesize that the low (and inconsistent) MeOH FE at low CoPc:MWCNT ratios could be due to CO_2_ outcompeting CO for active sites. Investigating this competition should be a priority for future study and could be accomplished via electrolysis of mixed CO and CO_2_ feeds. The CO electrolysis data shown in Figures S10 and S11 show that this approach is feasible.

Optimal conditions were not the goal of this study, but to show other selectivity controls that are important to CoPc catalysis. A design of experiments would be better suited to explore this wide parameter space if one wanted to optimize this catalyst. As also suggested in the Introduction, using PEDOT:PSS makes integration into solar cells easier and can also be the subject of future work.

## Conclusions

In summary, we have demonstrated, using a simple, and PFAS free-preparation of CoPc/MWCNT catalysts, the large impact of hydrodynamics on CO_2_R selectivity to MeOH. Electrodes were prepared by coating an Au substrate with PEDOT:PSS followed by a layer of the CoPc/MWCNT. Low electrolyte convection was shown to favor HER, while high electrolyte convection favors CO_2_ reduction to CO. An intermediate, optimal inlet flow rate of 8.5 cm/min leads to a high MeOH FE of 36% (±3%) at −1.2 V versus RHE, which suggests that CO is an unbound intermediate in the formation of MeOH. These experimental results match qualitatively with a 2D continuum model of the system. The model shows that convection helps to keep CO localized to the catalyst surface but too large of an electrolyte velocity can cause CO to be swept out of the system before converting into MeOH. Our catalysts were also found to contain aggregates, showing that dispersion is not a necessary condition for high MeOH FE. The strain conditions of catalyst were difficult to interpret due to the catalyst’s low electroactive fraction. The definitive role of formaldehyde, which was observed in some cases, could be not be established.

## Data Availability

The raw data and STEP files of the electrochemical cells will be uploaded to a public repository upon publishing.

## Supporting Information Available

Detailed experimental methods, ^13^C-labeling experiments, GC–MS method details, list of model parameters, schematic of electrochemical cell, schematic of liquid flow and gas flow through the electrochemical reactor, ratio of CoPc to MWCNT to the Faradaic efficiency to methanol, CAs of control experiments, example of the gas Faradaic efficiency, ^1^H NMR spectra, ^13^C NMR spectra, current density with varying potential on CoPc/MWCNT catalyst, illustration of model domain and all the mathematical forms of the boundary conditions, surface plot of CO concentration, XPS spectra, CV of the Au/PEDOT:PSS/MWCNT:CoPc electrode, and example chromatogram of a sample containing formaldehyde after derivatizing with PFBHA (PDF)
