# Supplementary material for: Role of Mass Transport in Electrochemical CO2 Reduction to Methanol Using Immobilized Cobalt Phthalocyanine
Source: ACS Appl Energy Mater. 2024 Apr 4;7(8):3091–8. doi: 10.1021/acsaem.3c02979 (PMC11040529; doi:10.1021/acsaem.3c02979)
Supplement: Supplementary file 1 — ae3c02979_si_001.pdf [file ae3c02979_si_001.pdf]

Supporting Information:

Role of Mass Transport in Electrochemical CO_2 Reduction to Methanol Using Immobilized Cobalt Phthalocyanine

Thomas Chan[1,2,6,7,8,†], *Calton J. Kong*[1,2,4,†], *Alex J. King*[1,2,5], *Finn Babbe*[1,2],

Rajiv Ramanujam Prabhakar[1,2], *Clifford P.Kubiak*[6,7,*], *and Joel W. Ager*[1,3,4,*]

[1]Liquid Sunlight Alliance, [2]Chemical Sciences Division, [3]Materials Sciences Division, Lawrence Berkeley National Laboratory, California 94720, United States

[4]Department of Materials Science and Engineering, [5]Department of Chemical and Biomolecular Engineering, University of California, Berkeley, California 94720, United States

[6]Liquid Sunlight Alliance, [7]Department of Chemistry, [8]Department of Nanoengineering, University of California, San Diego, La Jolla, California 92093, United States

[†]Authors contributed equally

*Corresponding Authors. Email: cpkubiak@ucsd.edu, jwager@lbl.gov

Detailed Experimental Methods

Cell Fabrication

The fabrication of electrolysis cells with a 1 cm^2 active cathode and anode area is described here.

Two approximately 2 x 2 x 0.5 in PEEK blocks (McMaster Carr) were cut using a band saw. Each block was then placed in a mill (Bantam Tools, Othermill) where the liquid chambers, pilot holes where the screws are to be inserted were drilled, and the cell contours were cut. 1/8" bits were used for all cutting operations. The cell was then flipped over and face cuts were used to cut the cell to thickness (1 cm). The cells were then sanded either manually or with a belt sander to remove machine marks and to smooth the surface.

The pilot holes were then drilled completely using a 0.209" drill bit. Using a 0.043" drill bit, holes for the gas inlet and outlet were drilled at the ends of the flow lines.

For the cathode/anode chamber, a hole punch was used to make pilot holes above and below the chamber. A 0.128" drill bit was then used again to drill a hole for liquid flow. 2 cm segments of 1/8" OD PEEK tubing (Trajan) were then cut and inserted into the holes with epoxy as the adhesive.

On the catholyte chamber, on the center of another face, using a 0.063" drill bit, a thin hole was drilled for the reference electrode. Then a shallow hole was drilled in accordance to tap a ¼-28 hole, and the hole was then tapped using the ¼-28 tap. This is to fit a 1/8" flangeless PEEK fitting (Cole-Parmer), and a leakless Ag/AgCl reference electrode (eDAQ) was inserted into this fitting.

The back plates were prepared in the same manner, except using a 0.25" thick PC plate. Nature rubber gaskets (40A Durometer, McMaster Carr) were cut using a laser cutter. Long screws and wingnuts were used to hold the cell together.

See **Figure S1** for the geometry of the machined areas for the 1 cm^2 active area cell and for an overall schematic. Step files for the CAD drawings that were used to generate machine paths have been uploaded as additional supplementary materials.

Electrochemical Testing

The cell was assembled according to the schematic shown in **Figure S1** using a gasket to seal the layers (4 total). Cu tape was attached to the back of a carbon fiber paper anode, and the measured electrode. A cotton swab is then used to remove some of the catalyst coating from the top of the

electrode such that Cu tape can better adhere to it. The flow cell stack was order from bottom to top as followed: polycarbonate back plate, carbon fiber paper anode, rubber gasket, PEEK anode chamber, rubber gasket, SELEMION™ membrane, rubber gasket, PEEK cathode chamber, rubber gasket, tested cathode, and polycarbonate top plate. Upon assembly completion, the four screws were tightened with nuts to finger tightness. The Ag/AgCl reference is inserted last and screwed into side of the cathode chamber.

10 mL of 0.05 M K_2CO_3 were then poured into the anolyte and catholyte reservoirs. These reservoirs each had 4 inlets/outlets (gas in, gas out, liquid in, liquid out), though only the liquid in and out were used in the anolyte reservoir. Flexible tubing (Cole Parmer Masterflex 13) was used to connect the cell, electrolyte reservoirs, gas source, peristaltic pump (Cole Parmer), and outlet to GC as shown in **Figure S2**. The gas outlet was connected to a 5 mL vial which was connected to the GC. This vial served as a mixing volume for the gases in order to time average the gas production since bubble formation and bursting can lead to inhomogeneities in the apparent gas flow.

The catholyte chamber was sparged and saturated using CO_2 gas at a flow rate of 50 sccm for 15 minutes before electrolysis. After 15 minutes the flow rate was switched to 5 sccm, which will continue for the remainder of the experiment. The peristaltic pump was then set to the desired flow rate (given the cell dimensions, volumetric flow in mL/min = lateral flow speed in cm/min)

The cell was connected to a potentiostat (Biologic SP-300), and an EIS measurement was taken to determine the solution resistance. The solution resistance was then inputted into a manual IR compensation method and a chronoamperometry (CA) experiment was performed for 80 minutes with 85% IR compensation.

Detection of Gas Products

The outlet of the catholyte chamber is connected to the inlet of the GC (SRI Multi Gas 3) to allow for gas detection. The GC is equipped with a 2 mL sampling loop which will inject the gas every 16.5 minutes. Two columns (Hayesep-D) and Mol Sieve 13x were used to separate the gases, and an electronic valve was used to redirect the bulk of the CO_2 band as to not overwhelm the detectors. A methanizer + FID detector was used for CO detection, and a TCD was used for H_2 detection. Argon was used as the carrier gas.

Detection of Liquid Products

Liquid products were determined using ^1H NMR (Bruker 500 MHz) following procedures similar to those described by Chatterjee *et al.*[1] An internal standard of 50 mM DMSO, and 50 mM phenol in DI water was prepared. Each NMR tube was prepared with 400 uL analyte, 50 uL internal standard, and 50 uL D_2O. The 90° pulse width was obtained for each solvent system and the center of the water peak. A solvent suppression method was then used to obtain the spectrum using a 25 second delay time. The integration ratio between the MeOH peak and DMSO peak was used to calculate the MeOH concentration. Calibration of the internal standard was done using different standard solutions of MeOH and potassium formate.

Detection of formaldehyde requires further sample preparation. 900 µL of electrolyte sample was mixed with 900 µL of a 100 mg/L solution of PFBHA and then heated at 60 °C for 10 minutes. The solution was then injected into an Agilent 7890A GC-MS. A fritted glass liner was used to hold all the salts would precipitate out upon heating. Method details are shown in **Table S1**. Calibration was done by doing the same steps as above but with standard solutions of 0.5, 1, 3, 6, and 10 mg/L of formaldehyde.

Calculation of Faradaic Efficiency for Gas and Liquid Products

The following formula was used to calculate the gas product faradaic efficiency:

$$\frac{C_{prod} \cdot J \cdot n \cdot F}{i} \times 100, \qquad \text{(S1)}$$

where C is the concentration of the product as determined by the GC, J is the molar flow rate of CO_2 into the reservoir, n is the number of electrons to the product (2 for both H_2 and CO), F is Faraday's constant, and i is the average current at the time of injection. The current used was the time averaged current in a 3-minute window, which corresponds approximately to the total mixing volume of the system. To calculate the error, 6 gas measurements were taken over the course of the 80-minute experiment for each experimental run. The first gas measurement is excluded from analysis as it is taken at the start (t= 0 min). Each experimental condition was repeated in triplicate separate run with fresh samples. The error of the gas measurements was calculated from the standard deviation of the all the gas measurements from the experimental condition.

The following formula was used to calculate the liquid product faradaic efficiency:

$$C_{ref} \cdot \frac{I_P}{I_R} \cdot \frac{H_R}{H_P} \cdot \frac{nFV}{Q} \cdot \frac{5}{4} \times 100, \quad \text{(S2)}$$

where C_{ref} is the concentration of the reference, I_P and I_R are the integrations of the product and reference respectively, H_R and H_P are the number of corresponding protons on the reference and product respectively (6 for DMSO, 3 for MeOH), n is the number of electrons to product (6 for MeOH), F is Faraday's constant, V is the volume of electrolyte used for the experiment (10 mL typically), Q is total charge passed, and the factor of 5/4 is due to 400 uL of analyte being diluted to 500 uL for the NMR analysis.

The error of the liquid product Faradaic efficiencies were calculated from the standard deviation of the triplicate experimental runs.

Calibration of Reference Electrodes

An aqueous solution of 0.05 M $K_3Fe(CN)_6$ was made in a glass scintillation vial. A glassy carbon disk was used as the working electrode (0.4 mm diameter), and a graphite rod was used as a counter. A CV was done from -0.1 V to 0.5 V, starting at 0.2 V at a sweep rate of 50 mV/s. The well-behaved redox feature was compared against literature and was monitored for any electrochemical drift or shifts.

$^{13}CO_2$ Labeled Experiments

The experiment for using labeled gas was exactly the same except for as follows. 0.1 M $KHCO_3$ was used, $^{13}CO_2$ was used as the gas (Sigma-Aldrich 99.0 atom % ^{13}C, < 3 atom % ^{18}O), the electrolyte was first sparged with Ar for 20 minutes at 50 sccm, then 5 sccm of $^{13}CO_2$ was flowed for 12 minutes before starting the experiment. The electrolysis was run for 45 minutes at -1.2 V vs RHE at 8.5 mL/min flow rate.

Details of Continuum Modeling

Steady-state mass conservation and Fick's law of diffusion is used to solve for the concentration profiles of CO_2 and CO within the electrolyte boundary layer adjacent to the cathode surface (model is illustrated in **Figure S13**).

$$D_i \nabla^2 \boldsymbol{c_i} + \boldsymbol{u} \cdot \nabla \boldsymbol{c_i} = 0 \quad \text{(S3)}$$

where D_i and $\boldsymbol{c_i}$ are the diffusivity and concentration of species i, respectively, and $\boldsymbol{u}$ is the electrolyte velocity. The first term captures the transport of species by diffusion, and the second term describes transport by convection.[2] The diffusivities and other model parameters are provided in **Table S2**.

The velocity profile is calculated by the continuity equation and the Navier-Stokes equation for an incompressible, Newtonian fluid.[3]

$$\rho\nabla\cdot\boldsymbol{u} = 0 \tag{S4}$$

$$\rho(\boldsymbol{u}\cdot\nabla)\boldsymbol{u} = \nabla\cdot[p\boldsymbol{I} + \mu(\nabla\boldsymbol{u} + (\nabla\boldsymbol{u})^T)] \tag{S5}$$

where ρ and μ are the density and dynamic viscosity of water, respectively (used material property value for water within COMSOL at 20 °C), p is pressure, and $\boldsymbol{I}$ is the identity matrix. Equations S3-S5 were solved simultaneously within COMSOL Multiphysics 6.0 software with a triangular mesh that coarsens going from the catalyst surface to the bulk of the electrolyte. A sensitivity analysis on the mesh identified a minimum mesh size of 11842 domain elements and 642 boundary elements.

The boundary conditions are presented in **Figure S13**. In short, a no slip condition is used at the catalyst surface, the bulk electrolyte boundary is treated as an open boundary, and inflow and outflow conditions were used at the entrance and exit, respectively. The surface reactions were modeled with simple first-order rate expressions where the rate constants (k_1 and k_2) were fit so that the simulated methanol current density versus inlet flow rate peaks around the experimentally measured optimal inlet flow rate (~ 8.5 cm min^{-1}). In general, changing k_1 and k_2 changes the inlet flow rate that maximizes methanol production.

Supplemental Tables

Table S1: GC-MS Method details

Instrument details	Agilent 7890A GC with 5975C MS and 7693 autosampler
Column	Agilent J&W DB-624 30 m (length) x 0.25 mm (i.d.) x 1.4 µm (film)
Column Flow	1.0 mL/min He at constant flow
Inlet Temperature	250 °C
Injection Volume	0.5 µL
Split Ratio	10:1
Oven Temperature	120 °C hold for 4 minutes, ramp at 30 °C/min to 150 °C, hold for 6 minutes, for a total of 11 minutes + 7 minutes post run at 200 °C
Mass Spectrometer	*Selected Ion Monitoring* (SIM) mode (m/z): 150, 161, 181, 182, 195, 198, 225 all at 50 ms dwell time<br>*Source temperature*: 230 °C, Quad temperature: 150 °C<br>*Solvent Delay*: 3.50 minutes

Table S2: List of model parameters.

Parameter	Value	Units	Ref.
$\boldsymbol{D_{CO2}}$	1.92×10^{-5}	$cm^2\ s^{-1}$	[4]
$\boldsymbol{D_{CO}}$	2.03×10^{-5}	$cm^2\ s^{-1}$	[4]
$\boldsymbol{L_{catalyst}}$	1	cm	–
$\boldsymbol{L_{electrolyte}}$	5	mm	–
$\boldsymbol{k_1}$	$\frac{k_2}{10}$	$m\ \ s^{-1}$	assumed
$\boldsymbol{k_2}$	2×10^{-5}	$m\ \ s^{-1}$	fit

Supplemental Figures

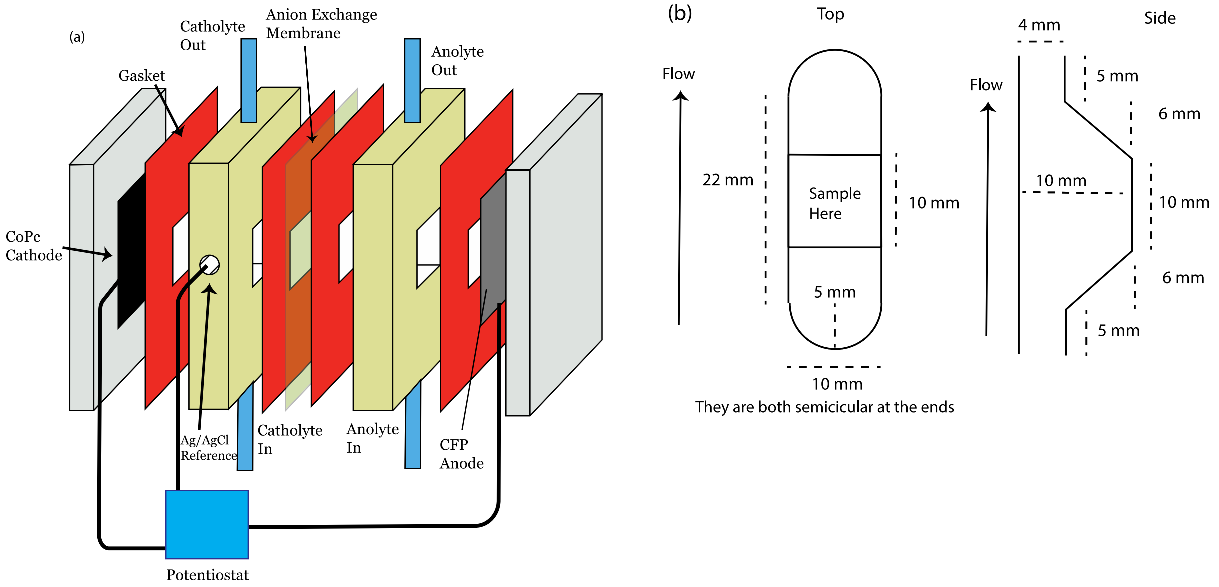

Figure S1: Schematic of electrochemical cell, and the geometry of the anode and cathode chambers.

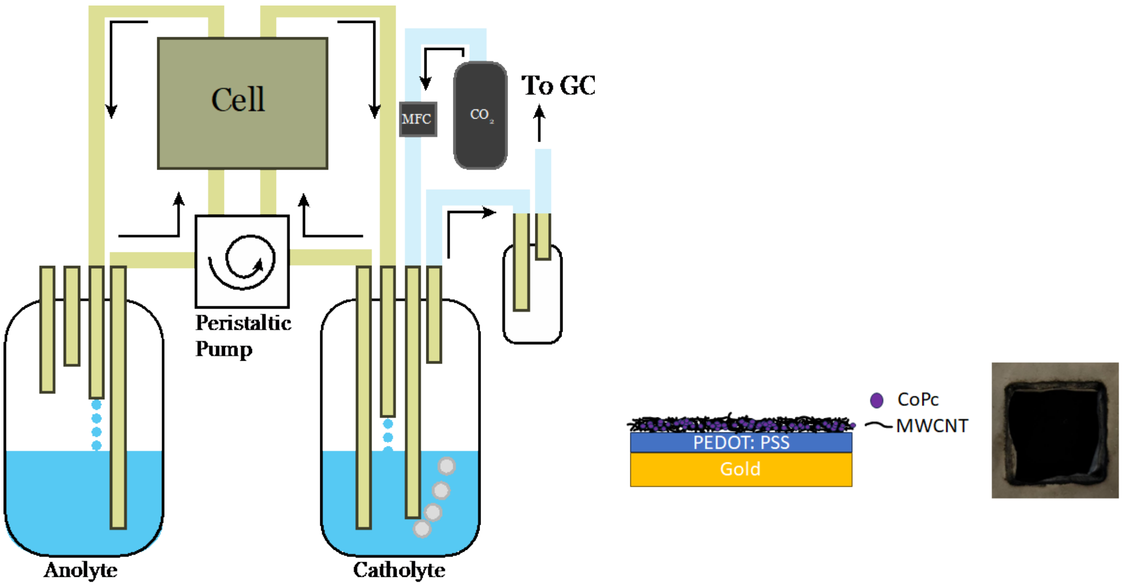

Figure S2: Schematic of liquid flow and gas flow through the electrochemical reactor and a schematic of the electrode stack along with an image of the electrode.

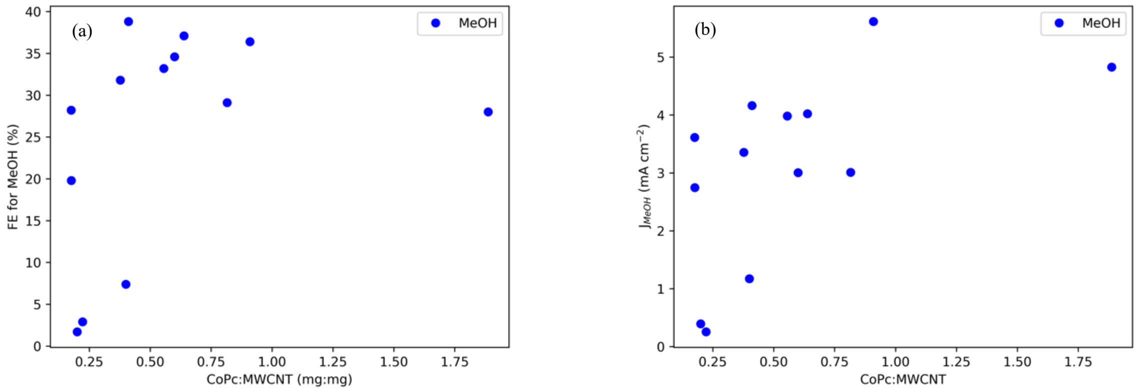

Figure S3: (a) Ratio of CoPc to MWCNT to the Faradaic efficiency to methanol. (b) partial current density to methanol. All tests were done in CO_2 saturated 0.1 M $KHCO_3$ with a linear electrolyte flow speed of 8.5 cm/min at -1.2 V vs RHE.

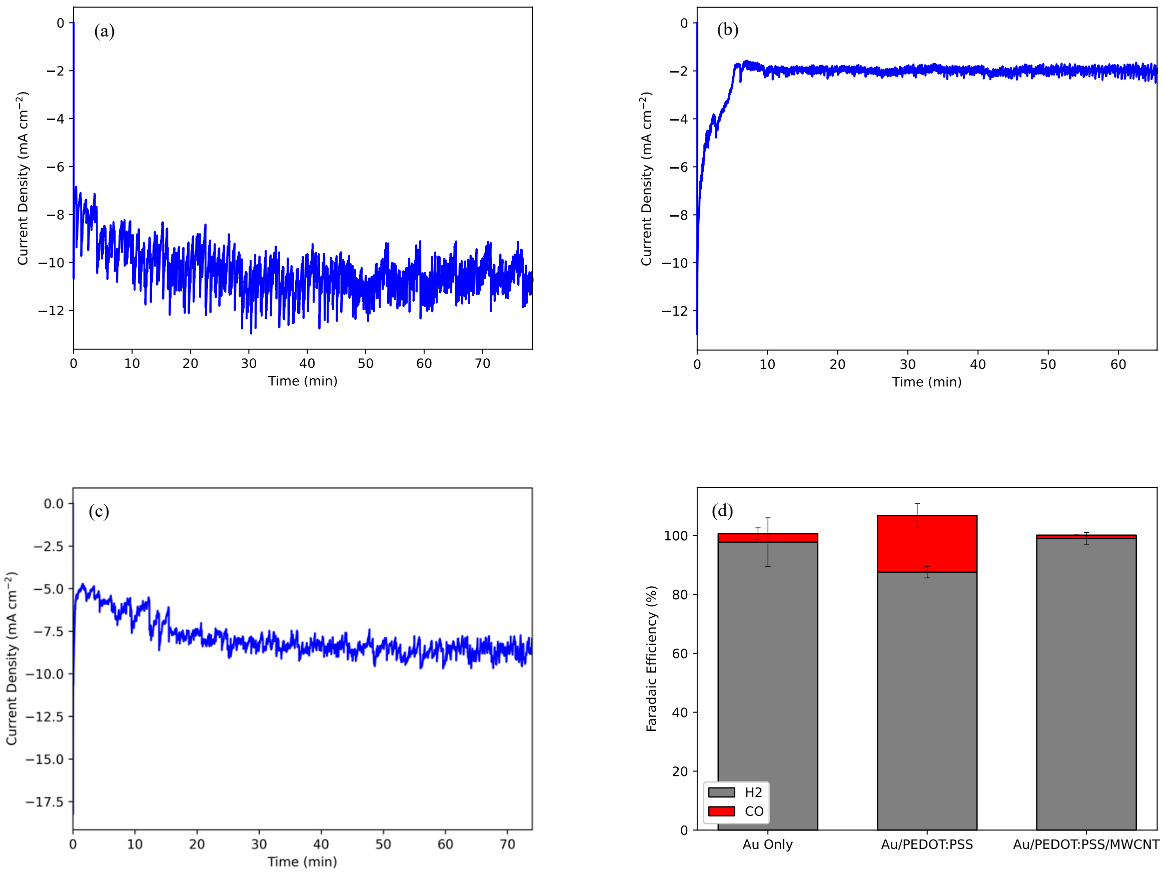

Figure S4: CAs of control experiments of (a) a bare, planar Au, (b) Au with PEDOT:PSS, (c) Au with PEDOT:PSS and MWCNTs. (d) FEs of products in the control experiments. All tests were

done in CO_2 saturated 0.1 M $KHCO_3$ with a linear electrolyte flow speed of 8.5 cm/min at -1.2 V vs RHE.

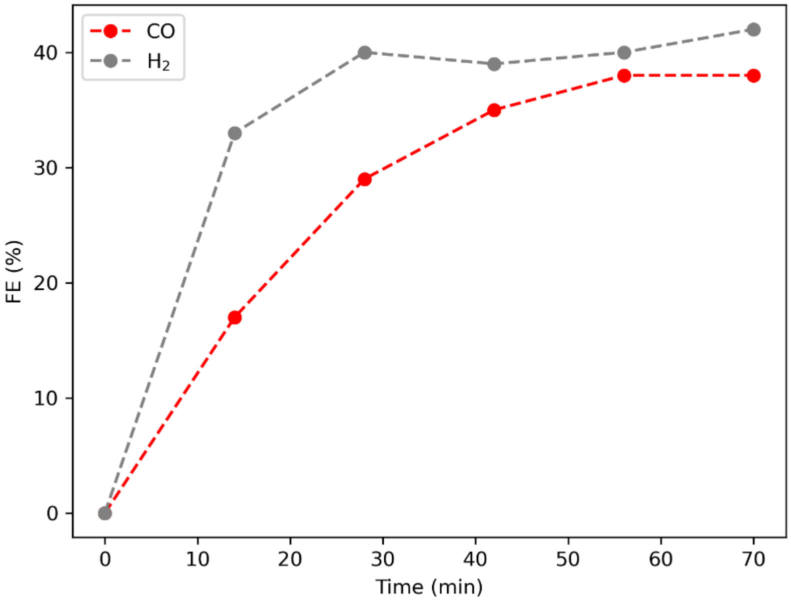

Figure S5: An example of the gas Faradaic efficiency over the course of a chronoamperometry experiment using CoPc/MWCNT at a 0.6:1 CoPc:MWCNT ratio. The gas Faradaic efficiency evolves during the first part of the experiment before reaching a steady state average. This electrode was held at -1.3 V vs. RHE in CO_2 saturated 0.1 M $KHCO_3$ with a linear electrolyte flow speed of 8.5 cm/min.

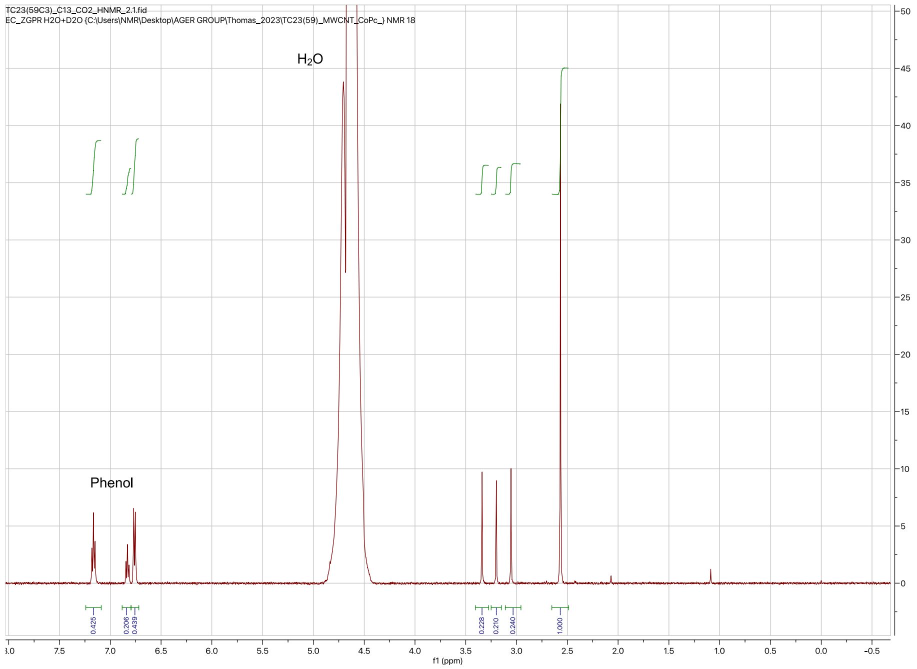

Figure S6: ^{1}H NMR of $^{13}CO_2$ labeled experiment showing proton coupling to the ^{13}C via splitting. $^{12}CH_3OH$ is present due to exchange of carbons with the $KH^{12}CO_3$ in the electrolyte. The J(C-D) coupling is 142.3 Hz. IPA presence is due to residual solvent in the ink.

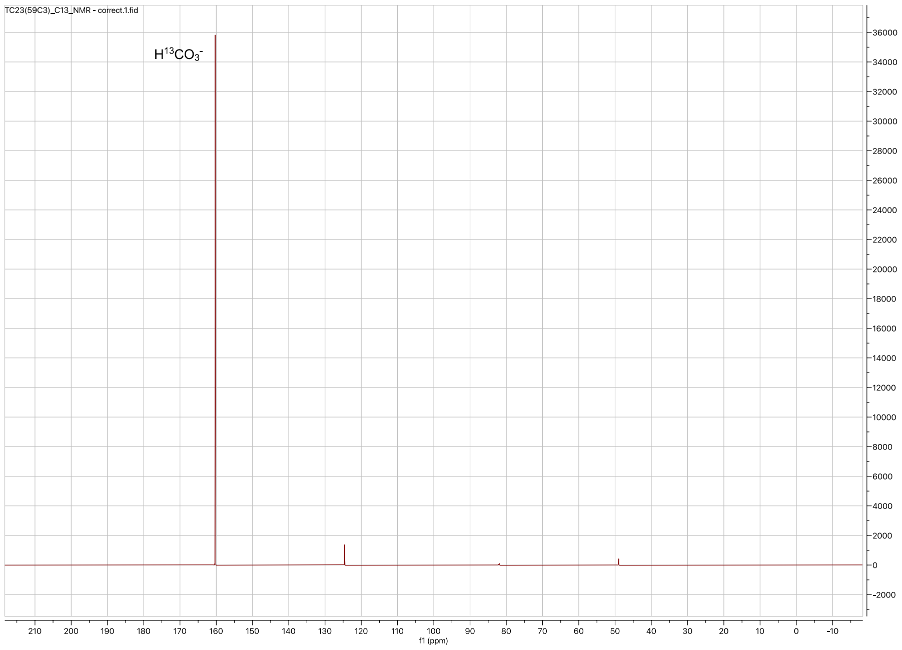

Figure S7: ^{13}C NMR of labeled experiment confirming that CO_2 is the source of methanol.

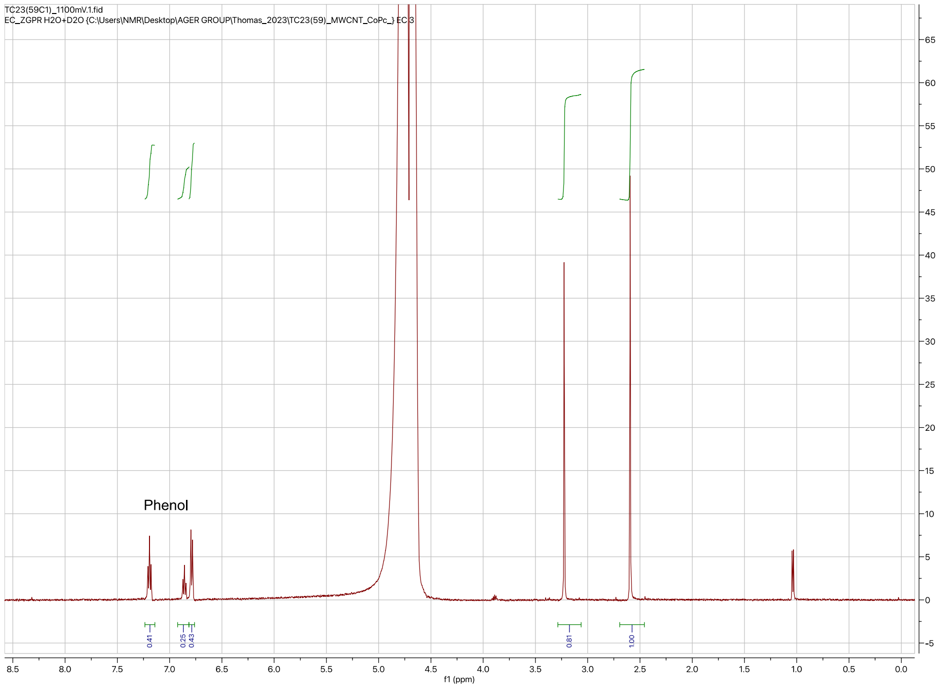

Figure S8: ^{1}H NMR of a control experiment with similar FE methanol as a reference for comparison with the $^{13}CO_2$ labeled experiment. The ^{1}H NMR shows that the predominate methanol species formed is ^{12}C based.

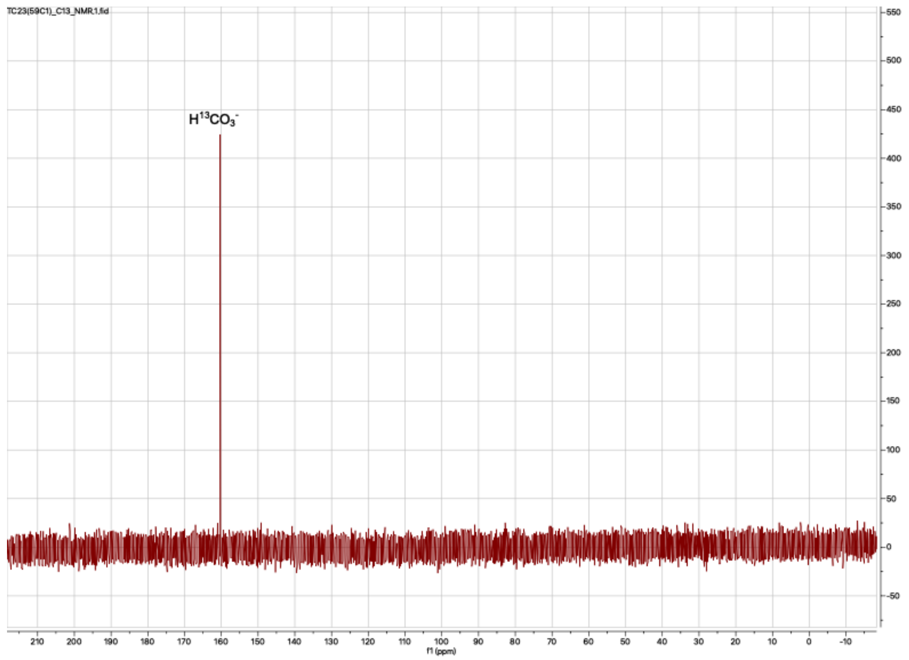

Figure S9: ^{13}C NMR of a control experiment with similar FE methanol as a reference for comparison with the $^{13}CO_2$ labeled experiment. This ^{13}C NMR shows that only ^{13}C labeled species is the $H^{13}CO_3^-$ which comes from sparging with the isotopically labeled $^{13}CO_2$.

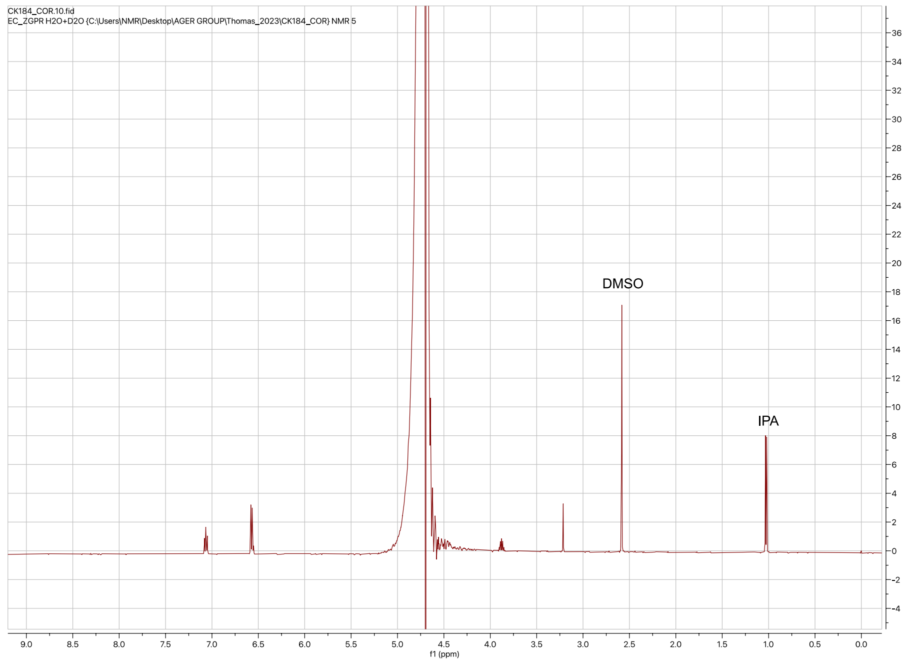

Figure S10: ^1H NMR of solution after CA using CO as the gas feed. Electrolyte was 0.1 M $KHCO_3$, electrolyte flow rate of 8.5 cm^3 min^{-1} with an applied potential of -1.8 V vs Ag/AgCl.

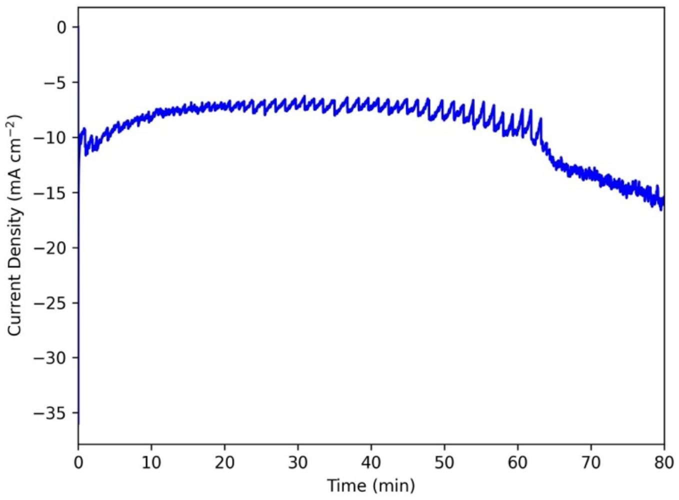

Figure S11: CA using CO as the gas feed. Electrolyte was 0.1 M $KHCO_3$, electrolyte flow rate of 8.5 cm^3 min^{-1} with an applied potential of -1.8 V vs Ag/AgCl.

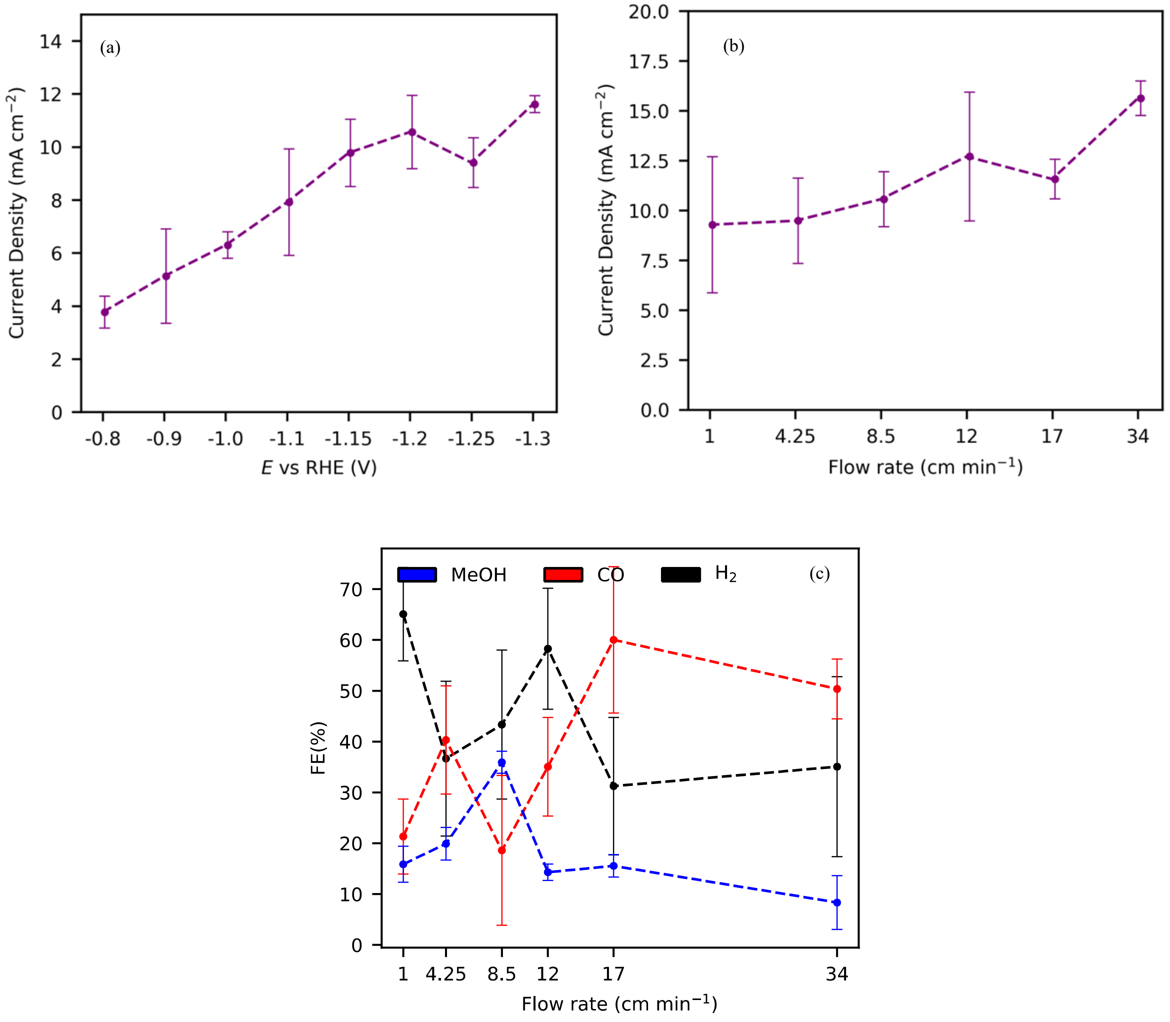

Figure S12: (a) Current density with varying potential on CoPc/MWCNT catalyst, (b) Current density with varying flow rate on CoPc/MWCNT catalyst. (c) Faradaic efficiency of the different products with flow rate, plotted individually. These corresponded with the main Figures 1 and 2 and were done with the 0.6:1 CoPc:MWCNT ratio at 0.15 mg cm^{-2} loading. Flow rate was kept constant (8.5 cm/min) when varying potential and potential was kept constant when varying flow rate (-1.2 V vs RHE).

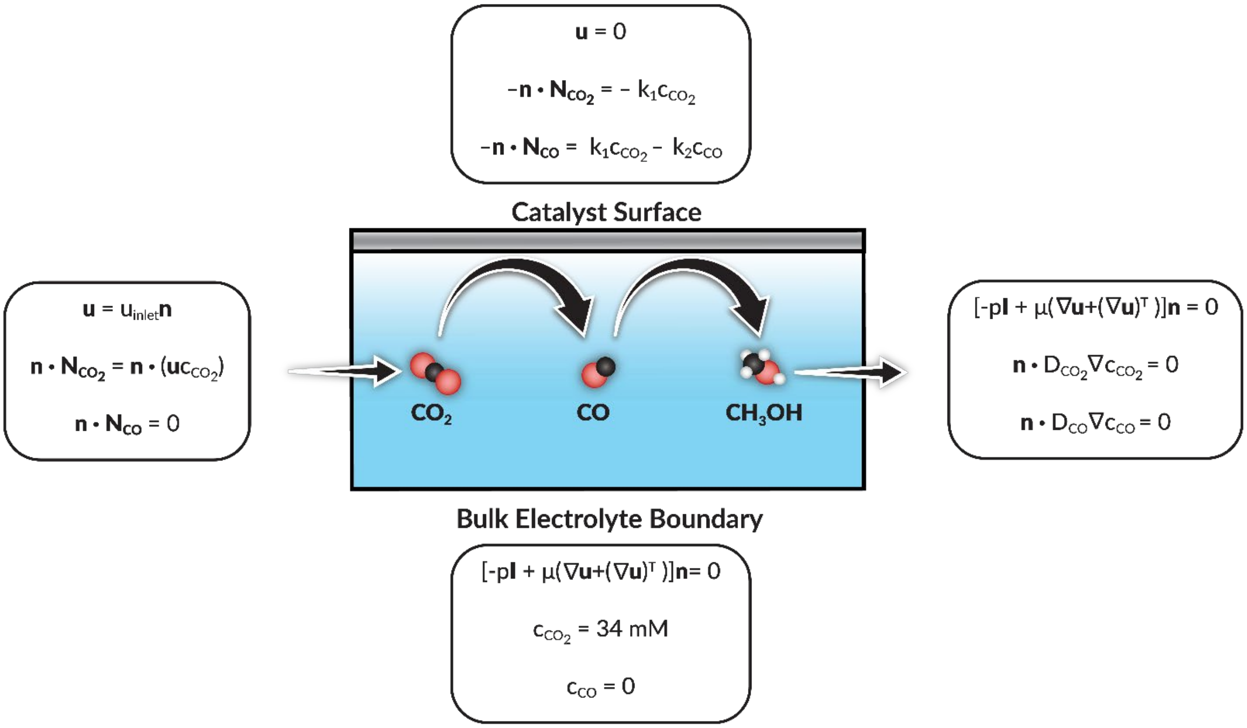

Figure S13: Illustration of model domain and all the mathematical forms of the boundary conditions.

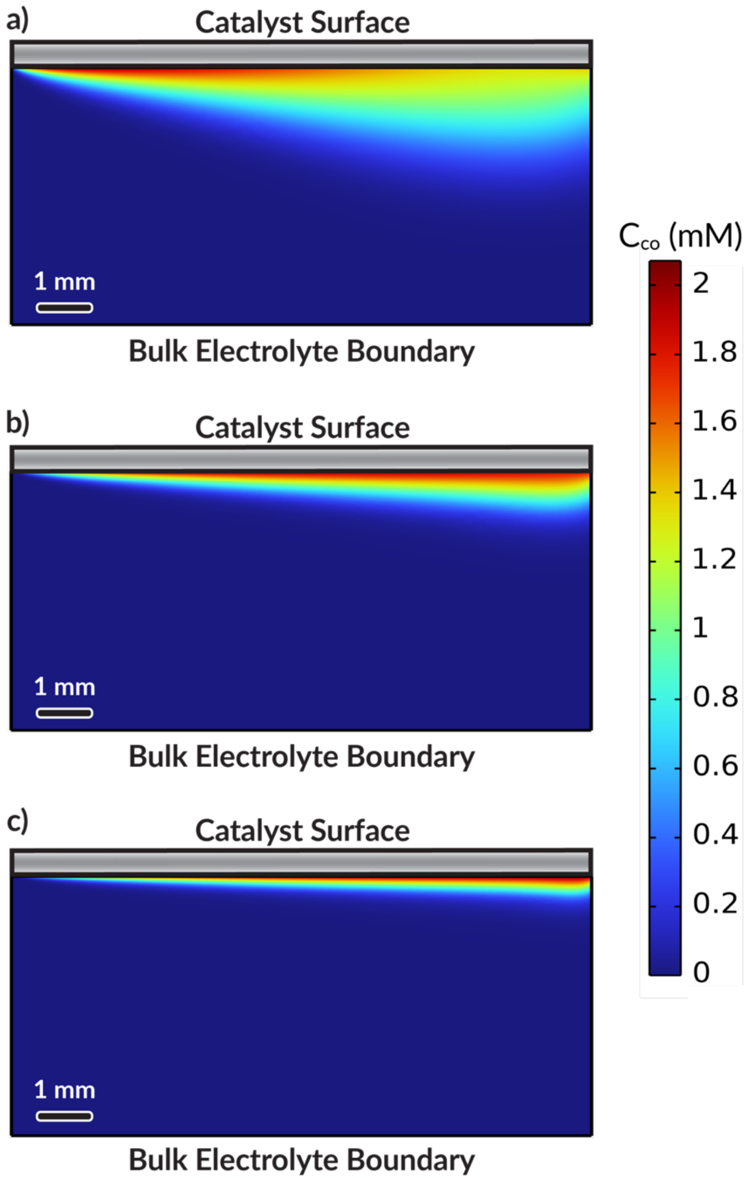

Figure S14: Surface plot of CO concentration throughout the model domain at an inlet velocity of **(a)** 1 cm min^{-1}, showing a case when lateral outward diffusion of CO limits efficient conversion **(b)** 8.5 cm min^{-1}, our optimized case where convection helps CO transport but not so fast as to sweep it away. **(c)** 30 cm min^{-1}; a case where convection dominates such that that CO is swept away before it can be further converted to methanol.

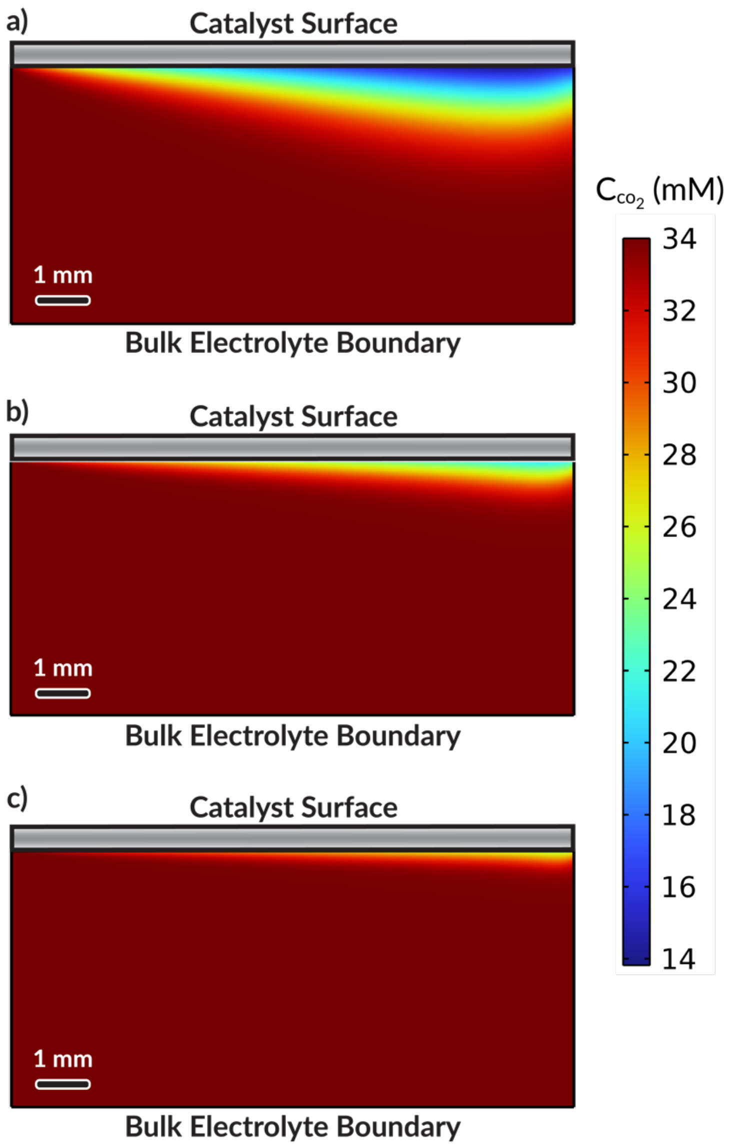

Figure S15: Surface plot of CO_2 concentration throughout the model domain at an inlet velocity of **(a)** 1 cm min^{-1}, showing a case when CO_2 depletion allows for stronger HER competition **(b)** 8.5 cm min^{-1}, our optimized case. **(c)** 30 cm min^{-1}

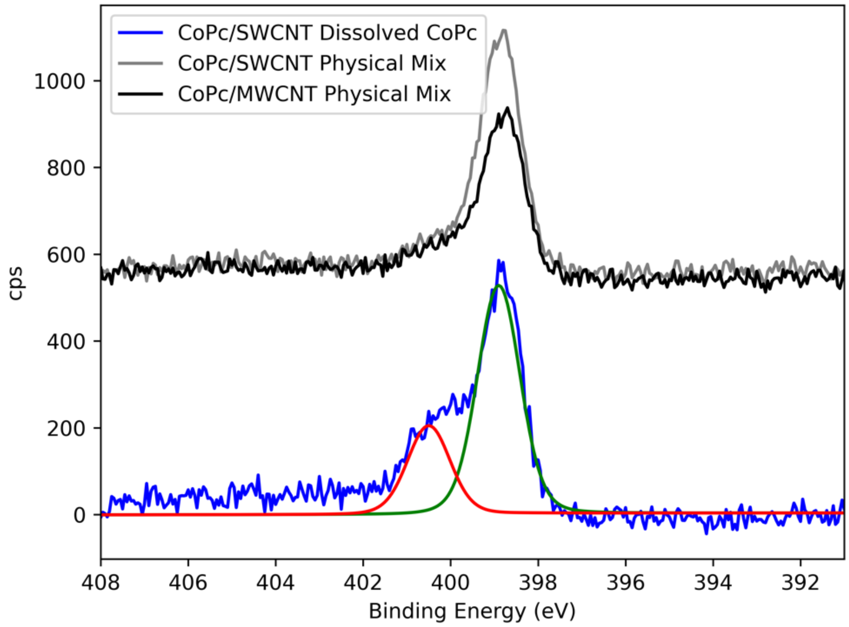

Figure S16: XPS core level spectra for N 1s for CoPc electrodes with different preparations: physically mixed MWCNT with CoPc, physically mixed SWCNT with CoPc, and SWCNT mixed with dissolved CoPc in DMF. The electrode prepared with dissolved CoPc clearly shows two separate peaks, providing evidence of strained CoPc in this case.

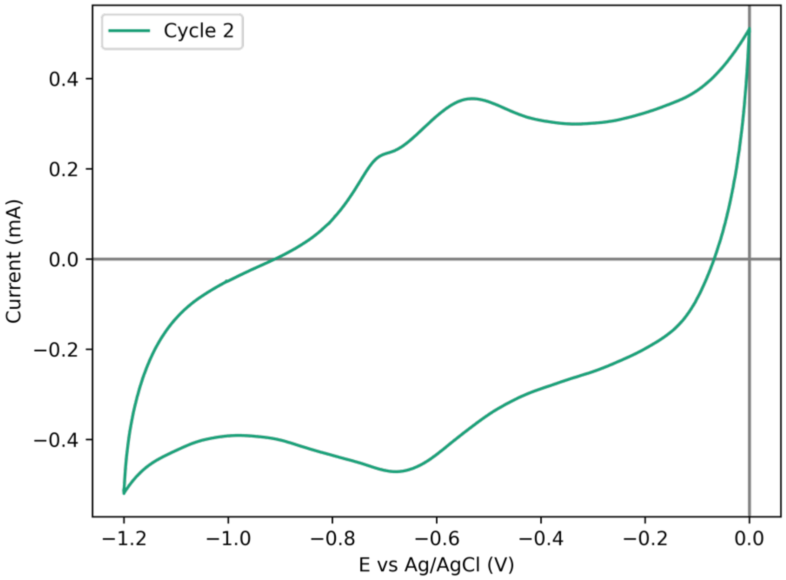

Figure S17: CV of Au/PEDOT:PSS/MWCNT:CoPc electrode at (loading 0.15 mg cm^{-2}) 20 mV/s showing the Co reduction feature. Integrating the peak gives us an electroactive fraction of 7%.

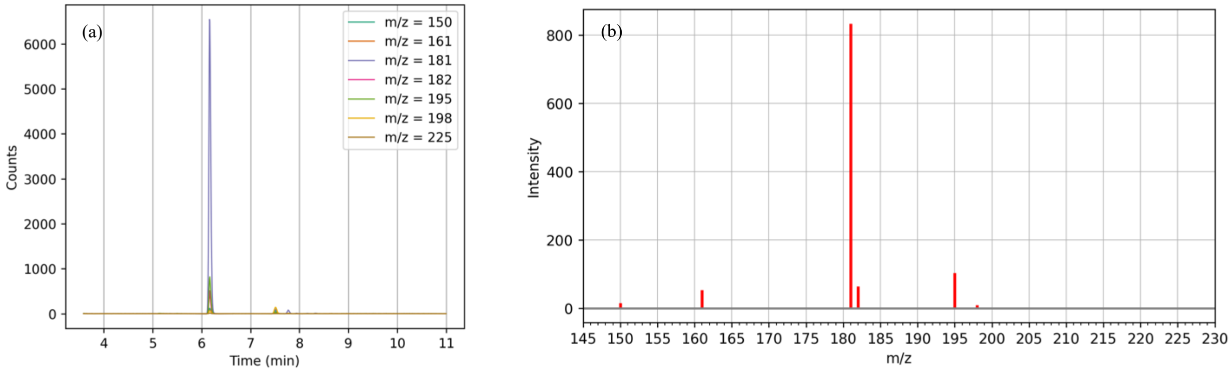

Figure S18: (a) Example chromatogram of a sample containing formaldehyde after derivatizing with PFBHA. The peak right after 6 minutes is the formaldehyde-oxime peak. The two peaks after are from the unreacted PFBHA. They elute as separate peaks due to equilibrium of the PFBHA with water. **(b)** Mass spectrum of the formaldehyde-oxime peak showing the selected ion masses.

Supplemental References

(1) Chatterjee, T.; Boutin, E.; Robert, M. Manifesto for the Routine Use of NMR for the Liquid Product Analysis of Aqueous CO_2 Reduction: From Comprehensive Chemical Shift Data to Formaldehyde Quantification in Water. *Dalton Transactions* **2020**, *49* (14), 4257–4265. https://doi.org/10.1039/C9DT04749B.

(2) Newman, J.; Thomas-Alyea, K. E. *Electrochemical Systems*, 3rd ed.; Wiley-Interscience: Hoboken, 2004.

(3) Welty, J. R.; Wilson, R. E.; Rorrer, G. L. *Fundamentals of Momentum, Heat, and Mass Transfer*, 6th ed.; Sayre, D., Ed.; Wiley-Interscience, 2014.

(4) King, A. J.; Bui, J. C.; Bell, A. T.; Weber, A. Z. Establishing the Role of Operating Potential and Mass Transfer in Multicarbon Product Generation for Photoelectrochemical CO_2 Reduction Cells Using a Cu Catalyst. *ACS Energy Lett* **2022**, *7* (8), 2694–2700. https://doi.org/10.1021/acsenergylett.2c01041.
